# Pneumopericardium Resulting From Blunt Thoracic Trauma

**DOI:** 10.7759/cureus.11625

**Published:** 2020-11-22

**Authors:** Rohan Anand, Steven E Brooks, MD, FACS, Yana Puckett, Robyn E Richmond, Catherine A Ronaghan

**Affiliations:** 1 Surgery, Texas Tech University Health Sciences Center, Lubbock, USA; 2 Trauma and Acute Care Surgery, Texas Tech University Health Sciences Center, Lubbock, USA; 3 Surgery, West Virginia University School of Medicine, Charleston, USA

**Keywords:** pneumopericardium, tamponade, blunt trauma, cardiothoracic

## Abstract

Pneumopericardium is a rare clinical condition defined by the presence of air in the pericardial sac. While this initially does not pose much danger, the accumulation of a sufficient amount of air can convert the pneumopericardium to a tension pathology. This may present with the classic signs, symptoms, and lethal dangers of cardiac tamponade. As with cardiac tamponade, treatment involves decompression of the pericardial sac through pericardiocentesis. This may be followed by insertion of a pericardial tube for continued drainage. While cardiac tamponade is well recognized by its classic findings, the rarer pneumopericardium may be more easily missed. This is further complicated by the backdrop of concurrent traumatic injuries in which it typically presents, as well as the absence of the defining accumulated pericardial effusion.

We present a case of a 38-year old male who developed pneumopericardium and worsening hemodynamic status as a complication to blunt trauma, a rare etiology for this condition. CT of the chest demonstrated air in the pericardium and a coexisting pneumothorax. A bedside chest tube was placed. Upon resolution of the pneumothorax, his hemodynamic status improved. Repeat bedside ultrasound demonstrated complete resolution of his pneumopericardium. This case emphasizes the importance of early recognition and diagnosis of this rare yet easily missed condition.

## Introduction

A pneumopericardium is defined as the presence of air or gas in the pericardial sac. It may be a simple pneumopericardium if the accumulated fluid is solely air or a complicated pneumopericardium if exudate or pus is additionally present [1]. Causes include congenital defects, thoracic surgery or other procedures, and positive pressure ventilation, particularly in neonates [1,2]. Most commonly, pneumopericardium is due to a fistula between the pericardium and a gas-containing cavity, such as the pleural space, trachea, bronchial tree, or GI tract [1]. This connection is often secondary to penetrating trauma creating a connection between the pericardium and the aforementioned structures. However, it is possible to have an isolated pneumopericardium with an intact visceral pleura. This occurs through the Macklin effect, where air tracks retrograde along the perivascular and peribronchial sheaths into the mediastinum [3,4]. Blunt trauma, most often due to deceleration injury from motor vehicle accidents or falls, is a much rarer etiology of pneumopericardium, with similar pathophysiological mechanisms and management as one due to penetrating trauma [2,5].

Pneumopericardium itself, while rare, is generally self-limiting and usually does not require further management aside from baseline monitoring. This includes electrocardiogram (EKG) and hemodynamic status. It may even be discovered incidentally during the workup for other traumatic injuries that were present, remaining asymptomatic until resolution. Symptoms, if present, are nonspecific, including dyspnea and precordial chest pain. Chest radiographs may depict an air-fluid level surrounding the cardiac shadow, though they may not show any changes at all. CT offers a better depiction of air in the pericardial sac and may disclose the source of the pneumopericardium as well. In a third of patients, however, a tension pneumopericardium may develop [1,5]. This presents with the clinical signs of cardiac tamponade but lacks the presence of effusion on radiographs. Such classic signs, however, may be easily missed due to the rarity of the condition in combination with concurrent severe traumatic injuries or comorbidities.

This article was previously presented as a meeting abstract at the 2020 North Texas Chapter of the American College of Surgeons (NTACS) Annual Meeting on February 21, 2020.

## Case presentation

**Figure 1:**
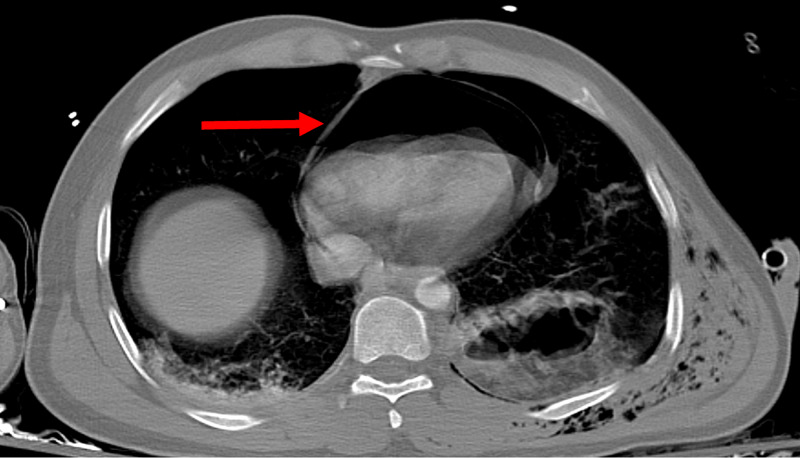
Transverse view of pneumopericardium, with the pericardium visible as a thin line bounding the air in the pericardial sac (red arrow). Mass effect on the left ventricle is present.

**Figure 2:**
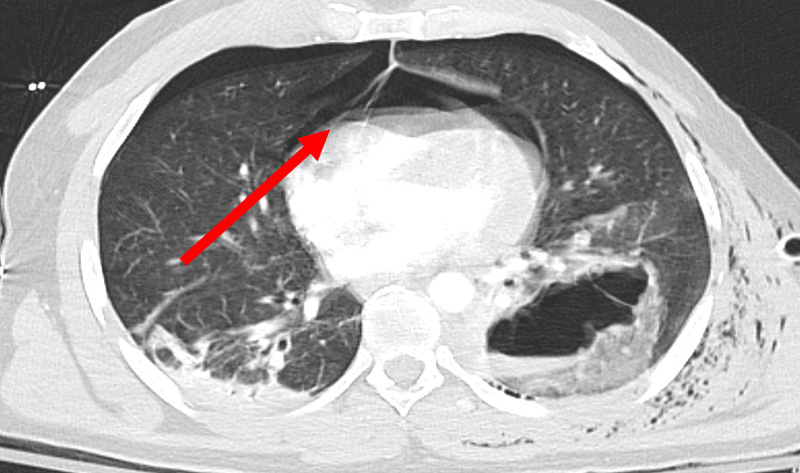
Transverse view of pneumopericardium, depicting a pericardial defect (red arrow).

A 38-year-old male with an unknown past medical history presented to the emergency department immediately following an unhelmeted motorcycle collision with a truck at highway speed. The patient was intubated at the scene, with the Glasgow Coma Scale calculated at 3T. Physical examination findings included a closed left femur fracture with crepitus, unstable pelvis, open left humerus fracture with crepitus, and left clavicle and scapula fracture. Additional findings included a left flail chest with chest wall subcutaneous crepitus and diminished breath sounds. A bedside 36-French left chest tube immediately drained approximately 100 mL of blood. CT revealed nondisplaced fractures of C7, T3, and the first rib. Bilateral pneumothoraxes, pneumomediastinum, and multiple areas of pulmonary hemorrhage and contusions were also visualized. Pneumopericardium was noted (Figures 1, 2), with a potential for evolution to tension pneumopericardium.

The patient was taken to the operating room for washout of orthopedic long-bone fractures. A 32-French thoracostomy tube was placed at the right fourth intercostal space. Upon entering the chest cavity, however, an audible gush of air was noted. Following placement of the chest tube and washout of orthopedic injuries, bedside cardiac ultrasound revealed complete resolution of his pneumopericardium. The patient remained hemodynamically stable. This suggested that the pneumopericardium may have been due to a communication between the right hemothorax and pericardium. Bronchoscopy and esophagogastroduodenoscopy (EGD) revealed no damage to the bronchial tree, esophagus, or stomach. The patient was then transferred to the intensive care unit (ICU) in stable condition, where he experienced an uneventful recovery. His chest tubes were removed 14 days post-admission, and he was subsequently discharged 19 days post-admission with the intent to follow up in one to two weeks in the clinic.

## Discussion

The first reported instance of pneumopericardium was in 1844 by Bricheteau [6]. He also first characterized the classical “bruit de moulin”, akin to the sound of water splashing on a mill wheel, which may be heard upon auscultation of the patient’s chest, owing to air and liquid in the pericardium. Common etiologies include excessive positive pressure ventilation or penetrating or blunt trauma. The two main pathophysiological mechanisms for the aforementioned etiologies include fistulas between air-containing cavities (such as the lung, bronchial tree, and gut) and the pericardium, and the Macklin effect. In the case of the former, and in the case of our patient, this most commonly presents as pneumothorax with disruption of the pericardium, allowing air from the pneumothorax to directly enter the pericardial sac. In the case of the Macklin effect, a rapid rise in intrathoracic pressure ruptures numerous alveoli, allowing air to escape into the interstitial lung space [3,4]. In the setting of an intact visceral pleural membrane, this air may dissect along the perivascular and peribronchial sheaths toward the lung hilum and into the mediastinum. This allows for the development of pneumomediastinum, which itself may additionally lead to pneumopericardium. A final, very uncommon, pathophysiological mechanism is gas production by an infectious source that has seeded the pericardium or any of the spaces in continuity with it.

Blunt trauma is an exceedingly rare cause of pneumopericardium. Capizzi et al. reported 32 such cases in the literature, mostly from motor vehicle accidents or falls [5]. Penetrating trauma, by its very nature, easily allows for the development of pneumothorax, pneumomediastinum, or pneumopericardium through fistula creation. Blunt trauma may cause any of these conditions as well. Capizzi et al. proposed the following three mechanisms by which blunt trauma may cause pneumopericardium: (1) a pleuropericardial connection in the setting of a pneumothorax, (2) the Macklin effect, and (3) a tracheobronchial-pericardial communication. Nevertheless, given the rarity of the condition, a higher index of suspicion is required to correctly diagnose such cases.

Pneumopericardium is generally a benign condition. Indeed, Shackelford considered the introduction of air into the pericardium to have little, if any, effect on the activity of the heart [7]. With sufficient introduction of air, however, a tension pneumopericardium may develop, with all the signs and dangers associated with an effusion-based tamponade. Adcock et al. in a series of controlled patient experiments demonstrated that raising the intrapericardial pressure from a normal baseline of 50-100 mmHg to 145 mmHg, equivalent to the addition of 60 mL of air, was sufficient to produce a rise in central venous pressure; at 265 mmHg, equivalent to the addition of 150 mL of air, hemodynamic derangement began to occur [8]. It is important to note that the rate of air accumulation is also an influential factor. A slower introduction of air allows the pericardium to adjust and accommodate as much as 500 mL of air, or even 1,000 mL of blood, without significant hemodynamic effect [9]. Nevertheless, the continued introduction of air, particularly amidst a delayed or missed diagnosis, increases the risk of a pneumopericardium developing into a tension pathology. Cummings et al. in a study of 252 pneumopericardium patients of various etiologies found that 37% of patients subsequently developed symptoms of tamponade, and the associated mortality was 56% [1]. Of interesting note, Capizzi et al. in their study of 32 pneumopericardium patients of blunt trauma etiology found that the same percentage of patients (12/32) developed tension symptoms [5]. This was most commonly associated with intubated patients (83.3%) or those with an associated pneumothorax (75%).

The symptoms of a tension pneumopericardium are almost identical to those of cardiac tamponade. Beck’s triad (hypotension, elevated jugular venous pressure, muffled heart sounds), pulsus paradoxus, cyanosis, and respiratory distress may be present. Additional symptoms include a low-voltage EKG and the bruit de moulin, though the latter is more likely to be heard in the case of a complicated pneumopericardium [10]. Hamman’s sign is useful to demonstrate the presence of subcutaneous air and may hint at the extent of air leakage. However, it is nonspecific to pneumopericardium and will only be readily audible if air has accumulated specifically over the vigorously contracting left ventricle [5]. Positive pressure ventilation may exacerbate the leakage of air and predispose the patient toward the development of tension pneumopericardium [1,5,11]. While the symptoms of tamponade are classical, they may easily be missed or obscured against a background constellation of symptoms in the typical polytraumatic presentation. As a wide range of etiologies may result in a hemodynamic collapse in such situations, pneumopericardium as the source of the hemodynamic instability may not be at the forefront of the differential until other sources have been ruled out, delaying the diagnosis and endangering the patient further.

Imaging is key to confirming the diagnosis. Chest X-ray (CXR) will classically show the cardiac shadow surrounded by air contained by the pericardium, visible as a thin, sharply delineated radiolucent line [2]. This classic finding was easily visible in our patient (Figures 1, 2). The heart itself may be compressed to a smaller size by the surrounding air, with removal of the air restoring normal heart size [7]. Importantly, the air will not rise above the level of the ascending aorta, which may be helpful in distinguishing the pneumopericardium from pneumomediastinum [10,12,13]. While posteroanterior CXRs may demonstrate air in front of the heart in pneumomediastinum, lateral CXRs will demonstrate radiolucency behind the sternum; pneumopericardium will demonstrate air in the pericardium only. Additionally, decubitus films will demonstrate immediate shifting of air in pneumopericardium as the patient shifts position in between films, whereas no such shift occurs in pneumomediastinum [14,15]

However, pneumomediastinum, pneumothorax, and pneumopericardium may coexist, furthering the diagnostic challenge; Cimmino additionally noted numerous instances in which pneumopericardium is, in reality, a misdiagnosed pneumomediastinum [13]. CXR may rarely even show a normal heart shadow with no evidence of air in improper places. When suspicion still remains, CT offers a more detailed examination of the pneumopericardium and may show the source of the air leak as well [15]. Bronchoscopy and EGD may also be useful to exclude a tracheobronchial or esophageal tear as the source of air, respectively.

Fortunately, when the etiology is known or elucidated, management of pneumopericardium is straightforward. In the case of a simple pneumopericardium, especially an asymptomatic one, it may resolve spontaneously and only require careful monitoring to ensure it does not transition to a tension pathology. If a tension pneumopericardium is present or develops, immediate decompression is indicated, typically by resolving the underlying cause. As in the case of our patient, a chest tube relieving a concomitant pneumothorax will by extension resolve the pneumopericardium. This is confirmed by repeat CXR or CT demonstrating a normal heart shadow with no air-fluid levels. Needle pericardiocentesis or percutaneous drainage is another option in cases where a thoracostomy tube does not resolve the hemodynamic instability. A more invasive option is a subxiphoid pericardial window, which may be created for further decompression and drainage of the pneumopericardium [7,16]. In the case of our patient, as the pneumopericardium was found to have resolved immediately upon chest tube placement, a subxiphoid window was not necessary. The placement of a pericardial tube as a precaution against the development of a tension pneumopericardium in the setting of a nontension pneumopericardium is debatable, and conservative treatment is typically sufficient [2,11].

## Conclusions

Pneumopericardium is a rare condition resulting from accumulation of air in the pericardial sac, typically secondary to penetrating trauma and rarely from blunt trauma. Treatment is indicated if tension pathology develops, consisting of immediate decompression of the heart and placement of a subxiphoid window for additional drainage as needed. In a patient with symptoms of tamponade but no visible effusion, particularly when other causes of hemodynamic instability have been ruled out, pneumopericardium should be suspected.
